# Net Survival of Elderly Patients with Gynecological Cancer Aged Over 75 Years in 2006-2008

**DOI:** 10.31557/APJCP.2019.20.2.437

**Published:** 2019

**Authors:** Shusaku Inoue, Hidemi Ito, Satoyo Hosono, Megumi Hori, Tomohiro Matsuda, Mika Mizuno, Kiyoko Kato, Keitaro Matsuo

**Affiliations:** 1 *Division of Cancer Epidemiology and Prevention, *; 3 *Division of Cancer Information and Control, *; 8 *Department of Gynecologic Oncology, Aichi Cancer Center Research Institute,*; 4 *Department of Epidemiology, Nagoya University Graduate School of Medicine,*; 5 *All Japan Labour Welfare Foundation Tokai Clinic, Nagoya,*; 6 *Department of Obstetrics and Gynecology, Graduate School of Medical Sciences, Kyushu University, Fukuoka, *; 6 *Division of Cancer Statistics Integration,*; 7 *Center for Cancer Registries, Center for Cancer Control and Information Services, National Cancer Center, Tokyo, Japan. *

**Keywords:** Survival- epidemiology, frail elderly, uterine neoplasms, ovarian neoplasms

## Abstract

**Background:**

The number of elderly patients with gynecological cancer in Japan is increasing in line with the aging of society. However, little has been reported on the survival of elderly patients aged 75 or older with gynecological cancer in Japan.

**Methods:**

To clarify survival in women aged 75 years or older with gynecological cancer, we analyzed data of 4,089 gynecological cancer cases (cervical cancer, 1,309 cases; endometrial cancer, 1,319 cases; and ovarian cancer, 1,461 cases) in patients aged 75 or older from 21 population-based cancer registries in Japan, diagnosed in 2006-2008. We calculated the net survival (NS) of younger (75-79 years old), older (80-84 years old) and the oldest age group (85-99 years old). We also calculated NS stratified by extent of disease and histological type.

**Results:**

Five-year NS of cervical cancer patients was 54.5% in the younger age group, 40.8% in the older age group and 28.2% in the oldest age group. Five-year NS of endometrial cancer patients was 64.5%, 51.6% and 39.0% in the younger, older and oldest age groups, respectively. Five-year NS of ovarian cancer was 34.7%, 18.8% and 8.3%, respectively.

**Conclusion:**

We estimated NS in elderly patients aged 75 years or older with gynecological cancers in Japan using data from population-based cancer registries.

## Introduction

A marked demographic change is ongoing in developed countries, including Japan, due to a low birthrate and increased life expectancy (Palaia et al., 2013; Arai et al., 2015). While the proportion of women in the global female population aged 75 years or older is 3.87% (Department of Economic and Social Affairs/Population Division, United Nations, 2015), the respective proportion in Japan in 2015 was 15.4%, double that two decades ago (Cabinet Office Government of Japan, 2013). Although total population has already peaked (Arai et al., 2015), the proportion of Japanese people aged 75 or older is estimated to increase 2.5-fold from 2010 to 2060 (Cabinet Office Government of Japan, 2013). More than 33,800 patients were diagnosed with gynecological cancers in 2012 in Japan (Nishimoto et al., 2016). Of these, 5,893 (17.4%) were aged over 75 years at diagnosis, while 3,492 (10.3%) and 1,760 (5.2%) were older than 80 and 85 years, respectively. These proportions are expected to rise even further over the coming decades as aging advances, leading to an increased burden of gynecological cancers among elderly women.

In addition to incidence and mortality, survival rate is also an important indicator in evaluating the burden of cancer. The trend to increasing gynecological cancer incidence in elderly women will increase the burden of these cancers, and in turn increase the importance of monitoring specific survival data of elderly patients with gynecological cancers. To date, however, this evaluation has not been actively pursued, either in Japan or worldwide. Most previous studies of treatment for gynecological cancer excluded elderly women aged 75 years or older (Susumu et al., 2008; Onda et al., 2016), or reported only one outcome measure for the whole age group (du Bois et al., 2003; Saito and Katabuchi, 2016). Thus, the applicability of these survival data to elderly patients seems unclear.

Here, to better understand survival among elderly patients with gynecological cancer, we analyzed data from population-based cancer registries in Japan.

## Materials and Methods

*Population*

We analyzed data of 4,089 patients aged 75 years and older from the Monitoring of Cancer Incidence in Japan (MCIJ) project who were newly diagnosed with gynecological cancer in 2006-2008. Data were collected from cancer registries in all 47 prefectures in Japan. Details of the framework have been described elsewhere (Matsuda et al., 2009; Hori et al., 2015). In brief, the Japan Cancer Surveillance Research Group has been collecting this data with the aim of estimating cancer incidence and survival in Japan. All subjects were followed for vital status in prefectural population-based cancer registries for at least 5 years after diagnosis by linkage to a death certificate database or the Basic Residential Registers. To warrant the validity of estimates, we selected 21 prefectures (Miyagi, Yamagata, Fukushima, Ibaraki, Tochigi, Gunma, Chiba, Kanagawa, Niigata, Fukui, Yamanashi, Aichi, Shiga, Osaka, Tottori, Shimane, Okayama, Hiroshima, Ehime, Nagasaki and Kumamoto) based on quality indices in the MCIJ database, namely 1) Death Certificate Only (DCO%: proportion of patients reported by DCO) of <25% or Death Certificate Notification (DCN%: proportion of patients first notified via death certificate) of <30%; and 2) Mortality to Incidence (M/I) ratio of <0.67 annually since 2006 for all cancers and all ages. The population covered in our study represented 47.1% of the total population of Japan in 2015. From this database we abstracted the data on patients aged 75 years or older with cervical cancer (ICD-10 code: C53), endometrial cancer (ICD-10 code: C54) and ovarian cancer (ICD-10: C56). We excluded patients with cancer of the uterus, NOS (ICD-10 code: C55) because detailed site information was not available, as well as those who were registered by death certificate only, in situ cases, those aged 100 years or older, and second primary cancer cases. Finally, 1,309 cervical cancer cases, 1,319 endometrial cancer cases and 1,461 ovarian cancer cases were included in the analysis.

*Statistical Analysis*

We estimated net survival (NS) using the Pohar Perme estimation method (STATA command ‘stnet’) (Perme et al., 2012; Coviello et al., 2015). This method is suitable for estimating survival in the hypothetical situation where the disease under study is the only possible cause of death. This estimation is made possible by decomposing the observed hazard of death into the hazard due to the disease (excess hazard) and that due to other causes (population hazard). The survival function derived from the excess hazard alone is termed the net survival (Perme et al., 2012). We used the complete national population life tables defined by single year of age and single calendar year to derive the background mortality of cancer patients (population hazard). We divided the patients into three categories, 75-79 years old (younger age group), 80-84 years old (older age group) and 85-99 years old (oldest age group). Patients were also classified into three clinical stages of extent of disease, namely localized (International Federation of Gynecology and Obstetrics (FIGO) 1988 stage 1), regional (FIGO 1988 stage 2-4a) and distant (FIGO 1988 stage 4b) disease groups. For histology, disease codes were based on the International Classification of Diseases for Oncology, 3rd edition (ICD-O-3) and patients were histologically grouped according to modified subgroups as defined in Cancer Incidence in Five Continents Vol. X published by the International Agency for Research on Cancer (IARC) (Ferlay and Rous, 2014). All data management and analyses were carried out using Stata SE Ver. 14.2 (StataCorp LP, College Station, TX). 

## Results

*Cervical cancer*

Characteristics and survival of the 1,309 subjects aged 75 years or more with cervical cancer are shown in Table 1. Among these, 39.3% were aged 75-79 years, 31.6% were aged 80-84 years and 29.1% were aged 85-99 years. With regard to the extent of disease, regional cases were the most prevalent (46.5%), followed by localized cases as the second-most prevalent (19.4%). Regarding histology, cases of squamous cell carcinoma and adenocarcinoma accounted for 68.2% and 13.8% of all cases, respectively. One- and 5-year NS of overall cervical cancer patients aged 75 years or older was 73.1% and 42.5%, respectively. One- and 5-year NS was 81.7% and 54.5% in patients aged 75-79 years; 71.8% and 40.8% in those aged 80-84 years; and 62.8% and 28.2% in those aged 85-99 years, respectively. By extent of disease, 1- and 5-year NS was 91.6% and 69.0% in the localized group, 79.5% and 46.1% in the regional group, and 33.5% and 6.3% in the distant group, respectively. By histology, 1- and 5-year NS was 79.3% and 50.8% in patients with squamous cell carcinoma, and 70.6% and 26.3% in those with adenocarcinoma, respectively. One- and 5-year NS of patients with cervical cancer stratified by age and extent of disease is shown in Supplementary Table 1.

*Endometrial cancer*

Table 2 presents the characteristics of the 1,319 subjects aged 75 years with endometrial cancer. Among these, 54.1% were aged 75-79 years, 29.0% were 80-84 years and 17.0% were 85-99 years. Regarding extent of disease, localized cases were the most prevalent (46.6%), followed by regional cases (20.0%). By histological type, endometrioid adenocarcinoma accounted for 49.4% and other specified adenocarcinoma cases, including serous adenocarcinoma or clear cell carcinoma, accounted for 11.8%. One- and 5-year NS was 77.5% and 56.3% in overall endometrial cancer patients aged 75 years or older; and 84.3% and 64.5% in those aged 75-79 years, 73.6% and 51.6% in those aged 80-84 years, and 62.3% and 39.0% in those aged 85-99 years, respectively. By extent of disease, 1- and 5-year NS was 96.0% and 84.8% in the localized group, 72.3% and 38.7% in the regional group, and 30.6% and 4.4% in the distant group, respectively. For histology, 1- and 5-year NS was 90.3% and 73.9% in patients with endometrioid adenocarcinoma, and 86.5% and 57.1% in those with other specified adenocarcinoma. One- and 5-year NS of patients with endometrial cancer stratified by age and extent of disease is shown in Supplementary Table 2.

*Ovarian cancer*

Table 3 describes the characteristics and survival of the 1,461 subjects aged 75 years or more with ovarian cancer. Among these, 44.6% were aged 75-79 years, 28.1% were aged 80-84 years and 27.3% were aged 85-99 years. With regard to the extent of disease, apart from cases of unknown extent, regional disease was the most prevalent, followed in order by distant and local disease. By histological type, unspecified malignant neoplasm was most prevalent, at 38.1%, followed by adenocarcinoma, not otherwise specified at 19.4%. One- and 5-year NS was 50.5% and 23.0% in overall ovarian cancer patients aged 75 or older; 68.4% and 34.7% in patients aged 75-79 years, 43.5% and 18.8% in those aged 80-84 years, and 28.2% and 8.3% in those aged 85-99 years, respectively. By extent of disease, 1- and 5-year NS was 96.7% and 84.8% in the localized group, 60.8% and 24.9% in the regional group, and 34.5% and 10.4% in the distant group, respectively. By histology, 1- and 5-year NS was 82.0% and 34.4% for serous carcinoma, 80.2% and 56.2% for mucinous carcinoma, 89.9% and 59.1% for endometrioid carcinoma, 81.3% and 62.9% for clear cell carcinoma, and 35.0% and 11.8% for other histological types, respectively. The 1- and 5-year NS of patients with ovarian cancer stratified by age and extent of disease is shown in Supplementary Table 3.

## Discussion

In this study, we performed a survival analysis of elderly patients with gynecological cancer using data from population-based cancer registries. Results showed that the NS of elderly patients was lower than that of all age patients (Saito and Katabuchi, 2016), and decreased in both age and disease extent-dependent manners. Moreover, elderly patients had more advanced disease. To our knowledge, this is the first study to evaluate survival among elderly patients with these cancers using data from registries in Japan.

In our study, disease stage in patients with cervical, endometrial and ovarian cancer was more advanced when compared to data for patients of all ages in a recent annual report of the Committee on Gynecologic Oncology of the Japan Society of Obstetrics and Gynecology (JSOG) (Saito and Katabuchi, 2016). Given that our present subjects were elderly patients aged 75 years or over, it may be unremarkable that the distribution by disease extent was more advanced than in other studies in patients of all ages. This trend has been reported elsewhere (Hoffman et al., 1995; Petignat et al., 2004; Fedewa et al., 2012). Among possible reasons, elderly women may have fewer opportunities to undergo gynecologic examinations, are less likely to undergo screening, and experience few specific symptoms that help localization of the diseased organ (Pignata and Vermorken, 2004). In addition, the distribution by disease extent in our study was more advanced than that of patients aged 70 years and over in the JSOG registry system (Saito and Katabuchi, 2016). This difference in distribution might be largely explained by the difference in registered subjects. Generally, hospitals registered in the JSOG registry system are core centers which provide the most up-to-date cancer treatment. In contrast, subjects in population-based cancer registries consist of all patients in their particular geographical region, including terminal patients who do not undergo aggressive treatment in either general or specialized hospitals. Therefore, data in our study may have included more cases of distant disease than those from the JSOG registry system.

**Table 1:** Characteristics and 1- and 5-Year Survival in Cervical Cancer Patients Aged 75 Years or Older

	Number of subjects	%	1-year NS (95%CI)	5-year NS (95%CI)
Total	1,309	100	73.1 (70.3-75.6)	42.5 (39.1-46.0)
Age				
75-79	514	39.3	81.7 (77.9-85.0)	54.5 (49.4-59.3)
80-84	414	31.6	71.8 (66.8-76.1)	40.8 (35.0-46.6)
85-99	381	29.1	62.8 (56.9-68.0)	28.2 (21.7-35.2)
Extent of disease				
Localized	254	19.4	91.6 (86.2-94.9)	69.0 (60.0-76.3)
Regional	608	46.5	79.5 (75.6-82.9)	46.1 (40.9-51.2)
Distant	137	10.5	33.5 (25.4-41.7)	6.3 (2.6-12.3)
Unknown	310	23.7	63.0 (56.9-68.5)	29.9 (23.6-36.5)
Histology				
Squamous cell carcinoma	893	68.2	79.3 (76.1-82.1)	50.8 (46.4-55.1)
Adenocarcinoma	180	13.8	70.6 (62.7-77.1)	26.3 (19.0-34.2)
Others	236	18.1	51.6 (44.6-58.1)	23.5 (17.2-30.5)
Other specified carcinoma	26	2		
Unspecified carcinoma	27	2.1		
Sarcoma	1	0.1		
Other specified malignant neoplasm	9	0.7		
Unspecified malignant neoplasm	173	13.2		

NS, net survival; 95%CI, 95% confidence interval.

**Table 2:** Characteristics and 1- and 5-Year Survival in Endometrial Cancer Patients Aged 75 Years or Older

	Number of subjects	%	1-year NS (95%CI)	5-year NS (95%CI)
Total	1,319	100	77.5 (74.9-79.8)	56.3 (52.7-59.6)
Age				
75-79	713	54.1	84.3 (81.2-86.9)	64.5 (60.2-68.5)
80-84	382	29	73.6 (68.5-78.0)	51.6 (45.1-57.7)
85-99	224	17	62.3 (54.9-68.9)	39.0 (29.6-48.2)
Extent of disease				
Localized	614	46.6	96.0 (93.3-97.6)	84.8 (79.5-88.8)
Regional	264	20	72.3 (66.1-77.5)	38.7 (31.6-45.8)
Distant	142	10.8	30.6 (23.1-38.5)	4.4 (1.5-9.6)
Unknown	299	22.7	66.5 (60.4-71.9)	38.1 (31.2-45.0)
Histology				
Endometrioid adenocarcinoma	652	49.4	90.3 (87.3-92.6)	73.9 (68.9-78.2)
Other specified adenocarcinoma	156	11.8	86.5 (79.1-91.4)	57.1 (46.8-66.2)
Adenocarcinoma, NOS	155	11.8	72.5 (64.2-79.3)	50.6 (39.9-60.4)
Others	356	27	52.3 (46.7-57.6)	26.3 (20.7-32.2)
Other specified carcinoma	18	1.4		
Unspecified carcinoma	35	2.7		
Sarcoma	39	3		
Other specified malignant neoplasm	81	6.1		
Unspecified malignant neoplasm	183	13.9		

NS, net survival; 95%CI, 95% confidence interval, NOS, not otherwise specified

**Table 3:** Characteristics and 1- and 5-Year Survival in Ovarian Cancer Patients Aged 75 Years or Older

	Number of subjects	%	1-year NS (95%CI)	5-year NS (95%CI)
Total	1,461	100	50.5 (47.8-53.1)	23.0 (20.5-25.6)
Age				
75-79	651	44.6	68.4 (64.6-71.9)	34.7 (30.7-38.7)
80-84	411	28.1	43.5 (38.5-48.5)	18.8 (14.6-23.3)
85-99	399	27.3	28.2 (23.6-33.0)	8.3 (4.9-12.9)
Extent of disease				
Localized	151	10.3	96.7 (88.7-99.1)	84.8 (72.1-92.0)
Regional	415	28.4	60.8 (55.7-65.6)	24.9 (20.3-29.7)
Distant	389	26.6	34.5 (29.6-39.4)	10.4 (7.3-14.0)
Unknown	506	34.6	40.4 (36.0-44.9)	12.8 (9.4-16.7)
Histology				
Serous carcinoma	238	16.2	82.0 (76.0-86.6)	34.4 (27.5-41.4)
Mucinous carcinoma	112	7.7	80.2 (70.6-86.9)	56.2 (44.1-66.7)
Endometrioid carcinoma	72	4.9	89.9 (78.3-95.4)	59.1 (44.0-71.3)
Clear cell carcinoma	52	3.6	81.3 (66.4-90.0)	62.9 (43.9-77.0)
Others	987	67.6	35.0 (31.9-38.1)	11.8 (9.5-14.3)
Adenocarcinoma, NOS	283	19.4		
Other specified carcinoma	32	2.2		
Unspecified carcinoma	64	4.4		
Sex cord-stromal tumors	7	0.5		
Germ cell tumors	10	0.7		
Other specified malignant neoplasm	34	2.3		
Unspecified malignant neoplasm	557	38.1		

NS, net survival; 95%CI, 95% confidence interval; NOS, not otherwise specified.

Among our results, the 5-year NS of patients aged 75 or older with cervical cancer and ovarian cancer was 42.5% and 23.0%, respectively. The Surveillance, Epidemiology, and End Results (SEER) program (Howlader et al., 2017) and European Cancer Registry-based Project on Survival and Care of Cancer Patients (EUROCARE) (De Angelis et al., 2014) reported closely similar survival rates for patients aged 75 or older. For our patients with endometrial cancer, 5-year NS was 56.3%, comparable with the 62% rate in the EUROCARE study (De Angelis et al., 2014) but lower than that of SEER by more than 10% (Howlader et al., 2017). The difference in survival between our present data and those of SEER may be partly explained by the difference in the distribution of disease extent. While our localized cases accounted for only 46.6% of the total, these accounted for 54.9% in SEER. In addition, unknown cases accounted for 22.7% of our total versus only 9.8% in SEER (Surveillance, Epidemiology, and End Results Program, National Cancer Institute, 2017). Nevertheless, further effort to elucidate other possible factors underlying this survival difference and to improve the survival of elderly patients with endometrial cancer in Japan are warranted. According to our present age-specific analyses, an age gradient in NS of 25% or more was observed over the three age groups in each cancer. Namely, the older the patient, the lower the NS, as other studies have already suggested (Markman et al., 1993). In all age groups, survival of ovarian cancer patients was lowest while that of endometrial cancer patients was highest among patients with the three gynecological cancers. Endometrial cancer is generally recognized as having a favorable prognosis because most patients tend to be diagnosed at an early stage due to the early onset of symptoms (Wright et al., 2012). In contrast, ovarian cancer is furtive and no effective screening method has yet been established. The majority of cases are therefore diagnosed at an advanced disease stage (Bhoola and Hoskins, 2006). These characteristics may suggest the reasons for the poor prognosis of patients with ovarian cancer.

We also estimated NS stratified by the extent of disease. The largest gradient by extent among the three gynecological cancers was seen in endometrial cancer, with the lowest 5-year NS in the distant group (4.4%) versus the highest 5-year NS in the localized group (84.8%). Five-year NS of elderly patients with localized ovarian cancer was 84.8%, the same as that in patients with localized endometrial cancer and higher than that of patients with localized cervical cancer. Recent substantial advances in perioperative anesthesiological management and surgical management have enabled more elderly patients with comorbidities to undergo surgical therapy more safely. Accordingly, favorable survival may be found even in elderly patients with early detected ovarian cancer. With regard to distant groups, poorest survival was estimated in endometrial cancer patients (4.4%) while the most favorable was estimated in those with ovarian cancer (10.4%). This tendency is consistent with that in a previous study among all age patients (Saito and Katabuchi, 2016). Generally, most distant cases of gynecological cancers are given systemic chemotherapy. Ovarian cancer has long been sensitive to chemotherapy, which may have affected the most favorable survival seen in our present patients with this disease.

On analysis by histological type, survival among cervical cancer patients was more favorable in those with squamous cell carcinoma than in those with adenocarcinoma. Among endometrial cancer patients, survival in those with endometrioid adenocarcinoma was more favorable than that of patients with the other types. In ovarian cancer patients, survival in those with serous adenocarcinoma was the most unfavorable among the four major epithelial ovarian carcinomas. These tendencies are consistent with those in the whole age group by the JSOG’s Committee on Gynecologic Oncology (Saito and Katabuchi, 2016). 

Our study has several strengths. First, our focus on the survival of very elderly patients aged 75 years or older is unique, and thus provides important information for elderly patients with gynecological cancers and medical care providers in geriatric gerontology. In addition, we evaluated survival in a very large group of gynecological cancer patients from a general population which accounted for 47.1% of the total Japanese population. Furthermore, we estimated unbiased survival, namely net survival using the Pohar Perme estimator method, in the elderly patient population (Danieli et al., 2012; Perme et al., 2012; Pohar Perme et al., 2016). Relative survival estimated by the conventional Ederer 2 method is biased when patients with a mortality hazard of the cancer under study also have a high hazard of other causes, which is a common situation for elderly patient populations. In contrast, net survival is the only measure that does not depend on hazards due to other causes.

However, several limitations of our study also warrant mention. First, we could not conduct further factorial analyses for outcome. This is because data from population-based cancer registration do not contain detailed clinical information on prognostic factors, namely performance status and comorbidities, or treatment modalities. Additional factorial analysis will require the establishment of a linkage system with more detailed clinical information or other data. Second, ovarian cancer patients who are diagnosed histologically are limited to those able to accept initial debulking surgery or a biopsy with laparoscopy. Conversely, patients who are considered inoperable due to poor general condition tend to be histologically classified as having unspecified malignant neoplasms. Consequently, the outcome of patients with the four major epithelial carcinomas may be biased toward favorable survival while that of other cancer patients may be biased toward lower survival. Third, even though we used the latest available data in our analysis, the timeliness of cancer registration and patient follow-up in Japan still lags that in North American and northern European countries by 2-3 years, and our most recent data were from 2008. A new law on the Promotion of Cancer Registries took effect in 2016, and this should bring about an improvement in the quality of data, including timeliness, completeness etc.

In conclusion, we used population-based cancer registries to clarify NS in elderly patients aged 75 or older with cervical, endometrial and ovarian cancer in Japan diagnosed from 2006 to 2008. A more detailed survival analysis which takes account of not only age, disease extent, and histology but also treatment contents and general condition at diagnosis is warranted.

## Funding Statement

This work was supported by a Grant-in-Aid for Research for Promotion of Cancer Control Programmes from the Ministry of Health, Labour, and Welfare, Japan (H26-political-general-013, H27-political-spesified-005).

## Conflict of Interest statement

Dr. Mizuno has received lecture fees from Chugai Pharmaceutical Co., Ltd. and Ono Pharmaceutical Co., Ltd. The other authors have no conflict of interest.
